# How is organ transplantation depicted in internal medicine and transplantation journals

**DOI:** 10.1186/1472-6939-14-39

**Published:** 2013-10-02

**Authors:** Céline Durand, Andrée Duplantie, Yves Chabot, Hubert Doucet, Marie-Chantal Fortin

**Affiliations:** 1 Centre de recherche du CHUM, Hôpital Notre-Dame, Pavillon J.-A.-de-Sève, 2099 Alexandre de Sève Street, Montreal, QC H2L 2W5, Canada; 2 Bioethics Department, Université de Montréal, Downtown Station, P.O. Box 6128, Montreal, QC H3C 3J7, Canada; 3 Transplant and Nephrology Division, Centre hospitalier de l’Université de Montréal, Hôpital Notre-Dame, 1560 Sherbrooke Street East, Montreal, QC H2L 4M1, Canada

**Keywords:** Organ transplantation, Ethical issues, Medical journals, Transplantation journals, Thematic content analysis

## Abstract

**Background:**

In their book *Spare Parts*, published in 1992, Fox and Swazey criticized various aspects of organ transplantation, including the routinization of the procedure, ignorance regarding its inherent uncertainties, and the ethos of transplant professionals. Using this work as a frame of reference, we analyzed articles on organ transplantation published in internal medicine and transplantation journals between 1995 and 2008 to see whether Fox and Swazey’s critiques of organ transplantation were still relevant.

**Methods:**

Using the PubMed database, we retrieved 1,120 articles from the top ten internal medicine journals and 4,644 articles from the two main transplantation journals (*Transplantation* and *American Journal of Transplantation*). Out of the internal medicine journal articles, we analyzed those in which organ transplantation was the main topic (349 articles). A total of 349 articles were randomly selected from the transplantation journals for content analysis.

**Results:**

In our sample, organ transplantation was described in positive terms and was presented as a routine treatment. Few articles addressed ethical issues, patients’ experiences and uncertainties related to organ transplantation. The internal medicine journals reported on more ethical issues than the transplantation journals. The most important ethical issues discussed were related to the justice principle: organ allocation, differential access to transplantation, and the organ shortage.

**Conclusion:**

Our study provides insight into representations of organ transplantation in the transplant and general medical communities, as reflected in medical journals. The various portrayals of organ transplantation in our sample of articles suggest that Fox and Swazey’s critiques of the procedure are still relevant.

## Background

In the 1950s, the possibility of replacing a damaged organ with a healthy one became a reality with the first successful renal transplantations. A number of technologically-driven advances in biomedicine followed in the 1960s, including dialysis, life-support treatment, definition of death and, particularly, the first heart transplantation [1]. The multidisciplinary field of bioethics emerged partly in response to these developments.

In the early days, organ transplantation was considered a "desperate [remedy] for desperate patients" [2], p.1483. With the development of immunosuppressive therapies, however, the field rapidly expanded. Nowadays, organ transplantation is widely accepted, despite a number of unresolved ethical issues. Transplantation challenges several accepted boundaries between self and non-self, body and machine, life and death, giving and receiving. The entanglement of social, cultural, medical and economic factors highlights the inherent complexity of this procedure [3]. Two authors who tackled this complexity were Renée C. Fox and Judith Swazey. They were privileged "journeyers into the field, participant observers, and chroniclers . . ." [4], p.197. However, they eventually decided to leave the field for reasons stated in the last chapter of their seminal work *Spare Parts* (1992): the routinization of the practice; the overidealization of its potential results; the triumphalist attitude of professionals who believe "death is our enemy"; nonchalant attitudes regarding the complexities of gift exchange; the massive financial investment in transplantation as opposed to other types of health care; and a general reluctance to consider the inherent uncertainties in this area of medicine [4]. The concept of uncertainty refers to difficulties in diagnosing, treating and accurately predicting the evolution and prognosis of individual patients. According to Renée Fox, this could stem from a physician’s personal ignorance, limits in actual medical knowledge, or a combination both, and is a source of anxiety for the patient, the physician and society. Medical and scientific advances do not rule out uncertainty, but modify its content and create new areas of uncertainty that were not previously known [5,6].

In a previous study on portrayals of organ transplantation in Quebec newspapers between 1995 and 2008, we found a similar lack of questioning with regard to the practice of transplantation. Although there was not a lot of hype around transplantation per se, journalists tended to overemphasize its successful, positive aspects. The focus was generally on patients’ and close relatives’ perspectives: the uncertainty of being transplanted, the desperate waiting for an organ, the medical procedure, and successful outcomes, with transplanted patients going on to live full lives (e.g., through pregnancy and athletic exploits). These articles did not look at complications, adverse effects, organ rejection and graft failure. The only two ethical issues mentioned were the selection of patients for the waiting list and the allocation of organs [7]. The newspaper coverage tended to exaggerate the "miraculous" aspect of transplantation and emphasize successful outcomes, leading patients to seek transplantation at any cost. These findings echoed Fox and Swazey’s observations in the early 1990s.

We wondered whether portrayals of organ transplantation in medical journals would be similar to those in the popular press, and whether Fox and Swazey’s analysis continued to be relevant and appropriate. We therefore focused the present study on the vocabulary used to describe transplantation, the ethical issues raised, patients’ experiences and the theme of uncertainty in internal medicine and transplantation journal articles published between 1995 and 2008.

## Methods

We selected the internal medicine and transplantation journals according to their impact factor, since this generally reflects the quality, prestige and readership of the journal. The top ten internal medicine journals (*New England Journal of Medicine*, *JAMA*, *The Lancet*, *Annals of Internal Medicine*, *British Medical Journal*, *Annual Review of Medicine*, *Archives of Internal Medicine*, *CMAJ*, *Annals of Medicine* and *PLoS Medicine*) and the two most important transplantation journals (*Transplantation* and the *American Journal of Transplantation*) were chosen. We limited our choice to these two transplantation journals, because they publish articles on different solid organ transplantations and are highly influential in the field. They are also the journals of leading transplantation societies: The Transplantation Society (*Transplantation*)*,* and the American Society of Transplantation and the American Society of Transplant Surgeons (*American Journal of Transplantation*)*.*

We searched the PubMed database using the following keywords to retrieve articles published between January 1, 1995, and December 31, 2008: transplantation or graft AND organ or liver or kidney or heart or lung or pancreas. We chose to look at this time period for two reasons: (i) it marked the arrival of the latest immunosuppressive drugs (Tacrolimus and Mycophenolate) which helped decrease the incidence of acute rejection [8,9], and (ii) it coincided with the period of our previous study on portrayals of organ transplantation in Quebec newspapers [7]. We retrieved 1,120 articles from the internal medicine journals and 4,644 articles from the transplantation journals.

A random sample of 5% of all the articles was used in order to clarify our inclusion and exclusion criteria (see Table 1 for further details). Following this stage, all of the 1,120 articles published in the internal medicine journals were read by one of the three researchers, and inclusion and exclusion criteria were applied. Our final sample from the internal medicine journals consisted of 349 articles.

**Table 1:** Inclusion and exclusion criteria

**Inclusion criteria**	**Exclusion criteria**
Solid organ transplantation is the main topic (kidney, liver, heart, pancreas, lung)	Letters to the editor
	Images in clinical medicine
	Articles about:
	• organ donation;
	• surgical procedures;
	• mechanical heart devices;
	• stem cell, bone marrow, composite tissue and pancreatic islet transplants;
	• xenotransplantation.

For the purposes of comparison, a random sample of 349 out of the 4,644 transplantation journal articles was created in order to have the same number of articles as in the internal medicine sample. Two researchers read all 349 articles from the transplantation journals, and applied the same inclusion and exclusion criteria that were used for the internal medicine journal articles. When an article was rejected, it was replaced by the following article in the list. These articles were proportionally distributed among the two journals and according to the publication year.

The following characteristics were quantitatively analyzed: the organ type, the continent of origin of the corresponding author, and the article format (research/clinical trial, brief communication, editorial, etc.). Descriptive statistics were used to describe the characteristics of the articles and the chi-squared test was applied.

The articles were analyzed using the content and thematic analysis method described by Miles and Huberman [10]. A random sample of 5% from both the internal medicine and transplantation journals was used to develop our coding scheme. The main themes in our coding scheme were: the vocabulary associated with organ transplantation, ethical issues grouped according to Beauchamp and Childress’ four principles (autonomy, beneficence, non-maleficence and justice) [11], as well as patients’ experiences, and sources of certainty and uncertainty. If the article described a clinical trial or research study (retrospective study, case series, cohort study), only the introduction and conclusion were coded, since the methods, results and discussion were not pertinent to our analysis. The computer software QSR NVivo (version 8.0) was used for the qualitative analysis. Twenty randomly selected articles were coded to assess the rate of coding agreement (78%).

## Results

### Article characteristics

**Figure 1:**
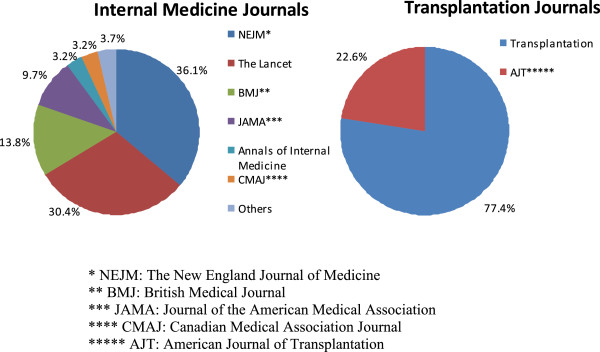
Article distribution.

Approximately two-thirds of the 349 articles retrieved from the ten internal medicine journals were from the *New England Journal of Medicine* and *The Lancet.* Among the 349 articles retrieved from the two transplantation journals, 77.4% were from *Transplantation*. There were fewer articles from *American Journal of Transplantation*, because it has only been in publication since February 2001 (see Figure 1 for the distribution of articles).

There was some variation between the internal medicine and transplantation journals in terms of the distribution of types of organ transplantation (p < 0.001). Renal transplantation was, however, the most common type in both journals (42.4% in the transplantation journals and 30.1% the internal medicine journals). The distribution of article formats also differed between the two types of journal (p < 0.001). There were more original articles (research and clinical trials) in the transplantation journals (63.3%) than in the internal medicine journals (31.8%). Conversely, there were more brief communications (news, reports, perspectives, sounding boards, essays) and editorials in the internal medicine journals (43.3%) than in the transplantation journals (20.6%) (see Table 2 for further details).

**Table 2:** Article characteristics

	**Internal medicine journals**	**Transplantation journals**	**P-value**
	**N = 349 (%)**	**N = 349 (%)**	
**Organs**			P < 0.001
Kidney	105 (30.1)	148 (42.4)	
Liver	72 (20.6)	87 (24.9)	
Heart	58 (16.6)	25 (7.2)	
Lung	26 (7.4)	14 (4.0)	
Pancreas	5 (1.4)	3 (0.9)	
Multi-organ	37 (10.6)	43 (12.3)	
Not mentioned	46 (13.2)	29 (8.3)	
**Continent of origin**			P = 0.3292
Americas	181 (51.8)	167 (47.9)	
Europe	135 (38.7)	154 (44.1)	
Other	33 (9.5)	28 (8.0)	
**Types of articles**			P < 0.001
Research, clinical trial	111 (31.8)	221 (63.3)	
Brief communication	110 (31.5)	69 (19.8)	
Review	46 (13.2)	23 (6.6)	
Editorial	41 (11.7)	3 (0.9)	
Case report	21 (6.0)	31 (8.9)	
Correspondence	17 (4.8)	2 (0.6)	
Other (patient’s testimony and guidelines)	3 (0.9)	0 (0)	

### Qualitative analysis

In this section, we will first present the vocabulary used to describe organ transplantation in the two types of journal articles analyzed. Our aim was to see whether organ transplantation was still portrayed as a routine treatment, as observed by Fox and Swazey. We will then discuss the ethical issues raised in the medical journals, grouped according to Beauchamp and Childress’ four principles mentioned above [11]. We will also present the patients’ experiences as reported in our sample. Finally, we will look at how uncertainty was addressed in our journal samples.

### Vocabulary used to describe organ transplantation

The vocabulary associated with organ transplantation in our article sample (similar in both the internal medicine and transplantation journals) fell into two main categories: the evolution of organ transplantation and organ transplantation as a routine treatment.

Most of the wording associated with the evolution of organ transplantation was very positive and even emphatic. The most frequently used terms were "success" (18 articles) and "improvement" (21 articles). The successes of organ transplantation were described as "remarkable," [12] "unprecedented," [13] "great" [14] and "striking" [15]. In approximately half of these articles, the words "success" and "improvement" were related to immunosuppressive drugs. The results of organ transplantation were seen to be synonymous with success and also with improvement and progress (8 articles): "Over the last 10 years, impressive progress has occurred in the 1-year patient and allograft survival rates after renal transplantation" [16].

The discovery of new immunosuppressive drugs and improved prevention of viral complications post-transplantation were associated with a "revolution" (7 articles) and were qualified as an "advance" (12 articles). The word "advance" was also used to describe all the improvements that have occurred in the field of transplantation, which have translated into positive outcomes for patients and organ survival: "Due to advances in organ preservation, surgical techniques and immunosuppressive agents . . . the 1-year patient and graft survival following organ transplantation have reached a zenith" [17].

One of the articles even qualified organ transplantation as "one of the most exciting medical advances in the late 20th and early 21st centuries" [18].

Although many advances and improvements have been made in the field of organ transplantation, which explain its successes, the practice is also described as a routine treatment (19 articles). It has become "commonplace," [19] "standard-of-care," [20] a "standard practice," [21] an "established" therapy [22] and an "accepted treatment" [23].

### Ethical issues

A total of 84 articles in the internal medicine journals (24.1%) and 23 articles in the transplantation journals (6.6%) addressed ethical issues. In the 84 articles from internal medicine journals, 44% were brief communications, including news, reports, perspectives, essays and sounding boards, whereas in the 23 articles from transplantation journals, 52.2% described research or clinical trials.

The ethical issues identified in our study can be grouped under the themes of autonomy, beneficence, non-maleficence and justice as described by Beauchamp and Childress (see Table 3 for quotations and further details). The theme of autonomy was discussed mostly in terms of informed consent, almost exclusively in the internal medicine journals. Except for a reference to informed consent in the research setting, there was no information on autonomy in the transplantation journals. In the internal medicine journals, the issue of informed consent was discussed from different angles: the consent of minors or incapacitated adults; patients who refuse transplantation; the information to be provided by the transplant team; and the consent of prisoner donors [24-27]. In the context of organ selling, the autonomy of buyers and sellers was discussed [28].

**Table 3:** Ethical issues

**Autonomy (almost exclusively in internal medicine journals)**	
**Informed consent: research, minors, incapacitated adults, refusal, information provided, marginal organs**	"Organs from donors with specified known infections may be considered for specific recipients—provided there is appropriate informed consent—based on the urgency of the need for transplantation and the availability of effective antimicrobial therapies" [29].
	"Consent is an important issue and it is clearly in the doctor’s interest to make sure that it is based on complete and explicit discussion of known risks" [30].
	"Hannah Jones persuaded Herefordshire Primary Care Trust that she was competent to make her own decisions about medical treatment and was making an informed choice not to have the operation that could have prolonged her life" [25].
	"A clear and comprehensive informed consent process is necessary, including a thorough and easily understood informed consent document. To control for conflicts of interest, informed consent should be obtained by independent third parties and should include counselling on all aspects of the risks and benefits of the experimental study" [31].
**Organ market: sellers’ and buyers’ autonomy**	"Paying for a kidney donation is viewed as a potential win-win situation that can benefit both parties. Individual decision making and patient autonomy have become the final arbiters of medical and bioethical values" [28].
**Beneficence (both types of journals)**	
**Risks vs. benefits: assessment methods, pregnancy (only in IM journals), transplantation with marginal donor, choice of medication, HIV patients**	"Physicians should discuss the risks and benefits of the various immunosuppressive regimens with respect to pregnancy and the fetus with their female patients and make decisions collaboratively" [32].
	"Adequate graft function requires lifelong immunosuppressive treatment, and the resultant modification of the immune system is associated with an increased risk of various cancers, particularly those involving viruses" [33].
	"In cases in which the need for transplantation is relatively less urgent, it is reasonable to avoid the use of organs from donors with unexplained fever, rash, encephalitis, or untreated infectious syndromes" [29].
	"This knowledge raised the intriguing possibility that the immunosuppression used to prevent rejection might be beneficial for patients with HIV disease that had been successfully controlled by antiretroviral agents" [34].
**Justice (both types of journals)**	
**Organ allocation**	"It is a relevant difference. You want to make sure you get the appropriate use of the appropriate resources. That is ethically not questionable at all. We do this rationalization of resources in health care every day" [35].
	"Because transplantable organs are scarce, determining the most ethical allocation system requires simultaneous considerations of efficacy, urgency, and equity" [36].
	"As stewards of a precious resource, the transplant community has a goal of achieving an equitable, transparent, and efficient system of organ allocation" [37].
**Disparities in access to organ transplantation (gender, ethnicity, geography)**	"We believe that the United States should end policies that permit geographic inequities and move quickly to determine the best use of data on the efficacy of outcomes to create a more equitable national system of distribution" [38].
	"The federal government should expect dialysis programs to meet reasonable standards for equitable access to transplantation for all patients, regardless of race, sex, or social status" [39].
**Personal responsibilities and organ transplantation**	"Even though the issue of personal responsibility for organ failure is usually raised in the literature with regard to whether patients who abuse alcohol should be given a lower priority for an organ, a similar argument could be made that a woman who wittingly chooses to get pregnant when her graft is unstable (and who therefore has an increased risk of graft loss) should be given a lower priority for retransplantation" [32].
	"A British liver transplant specialist last week accused colleagues of taking a decision on moral rather than medical grounds when they refused a transplant to a teenage girl with liver failure after she took half an ecstasy tablet" [40].

The themes of beneficence and non-maleficence, in both the internal medicine and transplantation journals, included an evaluation of risks and benefits. Two articles from the internal medicine journal sample also addressed the specific issue of pregnancy after renal transplantation (risks for the mother, graft and fetus) [32,41] and three articles discussed transplantation from high infectious risk donors [29,42,43]. Otherwise, risks and benefits were discussed in terms of: (i) predictable and individualized methods of risk assessment before registration on the waiting list [44,45]; (ii) risks and benefits of the transplantation [46-48]; (iii) choice of immunosuppressive drugs [49,50]; and (iv) transplants on patients with HIV [51,52].

Justice and fairness were the most commonly raised issues in our article sample (52 articles in the internal medicine journals and 19 in the transplantation journals). There was concern, for example, about the utility of transplantation and second transplantations [32,53]. In a similar vein, there was discussion about whether risk factors such as alcoholism should be taken into account in access to transplantation [54,55]. Also widely discussed was how gender, ethnic, geographic and socio-economic differences affect access to transplantation [37,56]. Young & Gaston noted that ". . . the median waiting time for a cadaveric kidney is almost twice as long (1185 vs. 605 days) for a black candidate as for a white one" [57].

Another issue raised was organ allocation and the organ shortage, which was described as a "huge and growing gap" [58] and "stark disparity" [59] between supply and demand which some felt would probably never abate [60]. There was a lot of uncertainty around what constitutes a sound organ allocation policy. Principles evoked included equity, equal chances, medical urgency, transparency, efficacy and decisions based on the disease causing organ failure. One article proposed hope (patients’ anticipation of being transplanted) as a principle of organ allocation [61]. Some articles also noted the size of the pool of potential recipients and questioned whether there should be an age limit for access to transplantation, and whether marginal donors should be allocated to older patients. The transplant community is held to high ethical standards—one article described its members as "stewards of a precious resource" [37].

### Patients’ experiences

A total of 18 (5.2%) articles from the internal medicine journals and 6 (1.7%) from the transplantation journals looked at patients’ experiences and feelings. Both positive and negative feelings were reported. Before transplantation, patients’ anticipation of the phone call announcing the availability of an organ was reported as a positive feeling [62]. Trust, well-being, increased energy, enthusiasm and gratitude for being alive were the positive feelings reported after the transplantation [63,64]. On the negative side, feelings of anxiety and depression were reported before and after the transplantation. Also noted was despair and anguish while waiting for the transplantation [65,66]. After the transplantation, there can be feelings of shame associated with non-compliance, as well as fear of rejection, frustrations and distress [67,68]. In addition to these findings, some articles reported mixed feelings such as "strength in weakness, hope in despair, wholeness in brokenness and life in potential death" [62] (see Table 4 for further details).

**Table 4:** Patients’ experiences

**Positive feelings**	"We jumped to answer each phone call, and our pager became the beeper of hope" [62].
	"Physically I was amazed at how well I held out. I felt really strong. My heart seemed to respond so well" [69].
	"With an organ transplantation, there is often a rebirth—a renewed awareness of the quality of life . . . It is not uncommon to see people pursue some dream which was put on a backburner because they were too busy or it was considered out of reach" [69].
**Negative feelings**	"At one point during the year and a half, I experienced a deep depression, thinking 'Why me’" [70]?
	"Not surprisingly, this can lead to anguish and acts of desperation for those who wait" [65].
	"Among the psychological variables it is above all anxiety, anger/hostility, and denial that may cause compliance problems. High level of anxiety was reported to be consistently the most important predictor" [67].
	"Most patients will not divulge nonadherence, not only because of shame and embarrassment, but also because admitting to nonadherence may make them less desirable candidates for retransplantation" [68].
**Mixed feelings**	"The past year has been painful and unforgettable. Our faith in God has been our greatest resource: we discovered strength in weakness, hope in despair, wholeness in brokenness and life in potential death" [62].

### Opposition between certainty and uncertainty

Feelings of uncertainty were equally present in the internal medicine journals (28 articles or 8%) and transplantation journals (37 articles or 10.6%) [61,71,72]. All aspects of organ transplantation were reported to be a source of uncertainty for patients and physicians: the quality of organ; the impact of organ transplantation on the patient’s quality of life; predictors of the patient’s and graft’s survival; the choice of treatment; possible complications and physiopathologic mechanisms. The only sources of certainty reported were graft loss in cases of non-compliance and death without transplantation [73,74] (see Table 5 for further details).

**Table 5:** Certainty and uncertainty

**Sources of uncertainty**	"The immediate implications of the data on ABO-incompatible heart transplantation in infants are uncertain" [71].
	"Information regarding their prognosis (i.e., predicted survival without transplant and recommendations for timing of listing are starting to emerge, although considerable uncertainty remains" [72].
	"Some patients may prefer early resolution of uncertainty to delay, especially if knowing the outcome of the allocation process changes their time horizon with respect to financial decisions" [61].
**Sources of certainty**	"There are a number of ways to approach diagnosis of the noncompliance syndrome, but the only certainty comes from direct patient admission of nonadherence to the prescribed immunosuppression" [74].
	"Therefore, whereas 20 years ago death was a near certainty without a transplant, and any length of survival after heart transplantation was regarded as a bonus, in the present era some patients potentially have a similar prognosis with alternative treatments" [63].

## Discussion

This is the first study to look at the non-biomedical aspects of organ transplantation reported in internal medicine and transplantation journals. These aspects include the language used to describe the procedure, bioethical issues, patients’ experiences and uncertainty. One strength of this study is the fact that we analyzed all the articles in the top ten internal medicine journals in which organ transplantation was the main topic, thus obtaining an accurate sense of how the practice is covered in this type of publication.

The terms used to describe the evolution of transplantation are generally very positive. Of course, organ transplantation is one of the major medical advances of our times. Patients whose condition was formerly a death sentence now have the promise of a new lease on life. In the words of Barbara Koenig, organ transplantation is part of the spectrum of end-of-life technologies since it is "deployed to stave off death" [75]. This positive emphasis is associated with the idea of progress. As Plough notes, progress in the West is associated with technological control and mastery of the body [76]. Organ transplantation in the case of organ failure is a prime example of this. Interestingly, at the same time as organ transplantation is described in terms of progress and scientific advances, it is also increasingly portrayed as a routine and commonplace treatment. Barbara Koenig defines a routine treatment as one that is not experimental or innovative; that is widely accepted in clinical practice; that involves typical hierarchical relationships between physicians, nurses, patients; and that has its own rituals. When a treatment becomes routine, physicians have a moral imperative to provide it and patients feel entitled to it [77]. The routinization of organ transplantation is no doubt one of the factors behind the ever-widening gap between the number of patients on the waiting list and the number of organs available. It is possibly also a factor behind the growing number of patients and families who launch public media appeals to plead their case for transplantation [78]. However, is it fair to describe organ transplantation as a routine treatment, considering all the issues involved in the transfer of an organ from one person to another [79,80]? How do we reconcile the highly technological and symbolic nature of organ transplantation with the fact that it has become the standard of care for patients suffering from organ failure?

As mentioned above, ethical issues (24.1% of internal medicine articles vs. 6.6% of transplantation articles) and patients’ feelings about transplantation (5.2% of internal medicine articles vs. 1.7% of transplantation articles) were reported more frequently in the internal medicine journals than in the transplantation journals. This can be explained by the different distribution of article formats in both types of journals. There were more editorials and brief communications in the internal medicine journals—formats that are more amenable to discussions of bioethical or social issues. One might conclude that it is not the role of transplantation journals to discuss social and ethical issues, since their aim is to publish recent scientific advances in the field of organ transplantation for physicians and scientists specialized in this field, whereas internal medicine journals target a wider medical audience. However, it is stated on the *American Journal of Transplantation* website that the journal’s scope includes ethical and social issues related to organ transplantation [81]. The journal *Transplantation* also has a special section (the Forum) devoted to ethical and controversial issues [82]. How to explain the contrast between these claims and scant coverage of ethical issues and patients’ experiences in our transplant journal sample? Should there not be more coverage of patients’ lives post-transplantation in transplantation journals, in order to remind transplant professionals that "life after a transplant is not like life before the disease [leading to transplant] entered the stage" [83]? Is there a discrepancy between the asserted scope of the journal and the editors’ choices of articles for publication? It is important to bear in mind that these two transplantation journals are those most read by professionals involved in the field. Given that the transplantation community is confronted on a daily basis with the practical problems of organ transplantation such as rejection and complications, has it become blinded to the non-biomedical aspects of the procedure? Does this reflect what Fox and Swazey described as the ethos of the transplant physician, which involves a heroic, pioneering, optimistic attitude and a refusal to accept limits [4]? Does it also reflect a division between transplant surgeons, who are mostly concerned with organ recipients, and internal medicine physicians, who focus on organ donors (living or deceased)? If we consider that ethical and social issues in transplantation do not have any legitimacy in scientific journals and should be addressed only in bioethics or social science journals, transplant professionals will have less to contribute to the ethical debate. Further studies are needed to explore these questions.

The ethical issues reported in the transplantation and internal medicine articles in this study can be grouped under Beauchamp and Childress’ four principles as described in the first edition of their seminal work, *Principles of Biomedical Ethics*[11]. This is not surprising, since their biomedical ethics approach is that most widely taught and applied in the medical field. However, we might question whether these four principles really capture all the ethical dimensions of organ transplantation.

Organ transplantation raises a number of ethical issues (living donation, gift exchange, incentives, etc.) which did not appear in our samples, most likely because we excluded articles on organ donation. This is one major limitation of our study. However, we wanted to focus on organ transplantation as we did in our analysis of Quebec newspapers. One of the most important ethical issues raised in the medical and transplantation journals was fairness in organ allocation, as it was in our sample of newspaper articles [7]. This could reflect the challenge of striking a balance between equity and medical utility in organ allocation policies. It is interesting to note that this issue has not been resolved in the more than 50 years that transplantation programs have been in existence [11].

Few of the articles in our sample looked at patients’ feelings about transplantation. In our previous study on portrayals of transplantation in Quebec newspapers, a significant number of articles described patients’ and their relatives’ perspectives on organ transplantation. The newspaper articles conveyed the despair of patients waiting for an organ and their return to a normal life following the transplantation. They also mentioned the transplanted patients’ exploits [7]. One might question whether both the newspapers and scientific articles examined truly conveyed transplant patients’ experiences. Previous studies on Irish renal transplanted patients showed that while they publicly described the benefits of the procedure and their enhanced well-being in order to show their gratitude for the gift of life, in private, they described their sickness, loneliness and mixed feelings about organ transplantation [3,80,84]. Is there a place for patients to give a frank account of their transplant experience and what success means to them?

A small number of articles in this study addressed the issue of uncertainty related to organ transplantation. In these articles, the entire process was described as uncertain, in contrast to what we found in our previous study on Quebec newspapers, where the uncertainty was related to whether or not the patient would receive an organ, but not to the outcomes of the procedure, which were invariably seen as positive [7].

Is there an overidealization of organ transplantation as described by Fox and Swazey? These two scholars referred to the tendency, in organ replacement, to overidealize the quality and duration of the life of transplanted patients, resulting in "seemingly limitless attempts to procure and implant organs" [4]. The positive vocabulary associated with the evolution of transplantation; the routine character of organ transplantation, which makes patients feel entitled to this treatment; the relatively few articles on the ethical issues associated with organ transplantation and on the real experiences of transplanted patients; and the portrayal of the organ shortage as an obstacle to be overcome could reflect an overidealization of the procedure and lead patients to seek transplantation at all costs (e.g., travelling to purchase an organ or publicly soliciting an organ via various media).

## Conclusions

Our study is the first to look at the non-biomedical aspects of organ transplantation (language, ethical issues, patients’ experiences) as portrayed in medical and transplantation journals. Our analysis shows that medical journal articles tend to describe the evolution of organ transplantation in positive terms and present it as a routine treatment. We can question whether this routinization of organ transplantation is related to the relatively small number of articles addressing ethical issues.

That said, organ transplantation was also described as uncertain in our sample of medical and transplantation journal articles. All aspects of organ transplantation are a source of uncertainty. This finding differs from that of our previous study on the portrayal of organ transplantation in Quebec newspapers, where the only uncertainty was related to the wait for an organ [7]. How to explain this difference in portrayals of uncertainty? Can the difference be attributed to certain journalistic practices aimed at creating "feel good" stories, [85] or does the greater number of success stories in newspapers reflect the medical and transplant community’s desire to promote organ donation [86]?

Although the organ shortage was one of the main ethical issues addressed in our sample of transplantation and internal medicine articles, it was rarely questioned. The medical community has a role to play in this shortage, since improvements in the field have led physicians to transplant patients who would previously not have been considered suitable candidates. The emphasis on the organ shortage in internal medicine and transplantation journals could imply it is a problem that can be solved and that all patients can be transplanted, including older and sicker patients. Is this a consequence of what Fox and Swazey, after working for decades in the field of organ transplantation, described as the "triumphalist" attitude of professionals in the face of death the enemy [4]? Does this emphasis on the organ shortage affect donor policies such as incentives, the acceptance of living anonymous donors, etc.? Further studies are needed to explore perspectives within the medical and transplant communities, as well as the general public’s views on the organ shortage and its relation to death, end-of-life care and health policies.

Would Fox and Swazey have been encouraged by the results of this study? Would they have considered returning to the field of organ transplantation? Their description of the routinization of the procedure and the ethos of transplant professionals characterized by an optimistic and heroic perspective are still present in the medical literature. The theme of uncertainty in organ transplantation is also present, but not to the same degree. The few articles that reported on patients’ experiences of organ transplantation presented some of the negative sides of the procedure. Finally, the articles analyzed in this study did not question the financial investment in organ transplantation compared to other forms of healthcare. Fox and Swazey’s criticisms are clearly still relevant, two decades later. Transplant professionals should be careful to maintain a critical stance with regard to the transplant process and should keep sight of the fact that it has moral, social and personal costs.

## Competing interests

The authors declare that they have no competing interests.

## Authors’ contributions

CD was involved in the data collection, content analysis, and drafting and revision of the manuscript. AD was involved in the study design, data collection, analysis and revision of the manuscript. YC was involved in the data collection, content analysis and revision of the manuscript. HD was involved in the study design, data interpretation and revision of the manuscript. MCF was involved in the study design, data interpretation, and drafting and revision of the manuscript. All of the authors have read and approved the final manuscript.

## Pre-publication history

The pre-publication history for this paper can be accessed here:

http://www.biomedcentral.com/1472-6939/14/39/prepub
